# Performance of Risk Stratification Tools for Cephalosporin Allergy Testing

**DOI:** 10.1001/jamanetworkopen.2026.34079

**Published:** 2026-09-16

**Authors:** Ami P. Belmont, Maren Valente, Aster Workineh, Samantha Novotny, Jason H. Kwah, Chang Su, Katelyn H. Wong, Elizabeth J. Phillips, Cosby A. Stone

**Affiliations:** 1 Section of Rheumatology, Allergy and Immunology, Department of Internal Medicine, Yale School of Medicine, New Haven, Connecticut; 2 Yale Center for Clinical Investigation, New Haven, Connecticut; 3 Division of Allergy and Immunology, Department of Medicine, Montefiore Medical Center, Bronx, New York; 4 Division of Pulmonary, Critical Care, Allergy and Sleep Medicine, University of California, San Francisco Medical Center, San Francisco; 5 Section of Pediatric Pulmonology, Allergy, Immunology and Sleep Medicine, Department of Pediatrics, Yale School of Medicine, New Haven, Connecticut; 6 Division of Allergy, Pulmonary, and Critical Care, Department of Medicine, Vanderbilt University Medical Center, Nashville, Tennessee

## Abstract

This cross-sectional study reports the validity of CEPH-FAST, Urticaria 1-1-1, and Vanderbilt criteria for use in patients with cephalosporin allergy.

## Introduction

Cephalosporin allergy labels (CALs) are common and limit use of first-line antibiotics.^1,2^ Given that most patients with CALs are not allergic, formal verification of these labels is critical for antimicrobial stewardship.^1,2,3^ Many studies have evaluated risk stratification to facilitate penicillin allergy evaluation and use of direct challenges, but few studies have focused on CALs.^4,5^ Studies have validated CEPH-FAST, Urticaria 1-1-1, and Vanderbilt criteria for CAL risk stratification.^6,7,8^ However, to our knowledge, none of these tools have undergone subsequent external validation.^6,7,8^ We aimed to examine the use and external validity of CEPH-FAST, Urticaria 1-1-1, and Vanderbilt criteria for cephalosporin allergy risk stratification.

## Methods

We performed a retrospective review of the medical records of patients with CALs evaluated at a Yale University–affiliated allergy and immunology clinic between December 1, 2014, to July 21, 2025. The Yale University Institutional Review Board deemed this cross-sectional study exempt from ethics review and waived the informed consent requirement because of secondary use of data. We followed the STROBE reporting guideline.

Under consecutive sampling, patients were eligible if they were 18 years or older and had undergone complete cephalosporin allergy evaluation. We defined complete evaluation as a consultative visit, followed by skin testing with drug challenge (if testing had a negative result) or direct cephalosporin challenge. Consistent with a guideline-recommended testing approach^1^ and the original CEPH-FAST study,^6^ we included only patients who had a cephalosporin challenge with the implicated cephalosporin or a cross-reactive cephalosporin. Excluded patients had a negative skin testing result but did not undergo a cross-reactive cephalosporin challenge to ascertain tolerance. A positive skin test result or an allergist-adjudicated immune-mediated allergic reaction was considered a verified cephalosporin allergy. We recorded whether each tool could be scored using allergists’ documentation.

Additionally, we calculated tool performance characteristics (eg, sensitivity, specificity, negative predictive value [NPV], positive predictive value [PPV], area under the receiver operating characteristics curve [AUROC]). Because intravenous (IV) cephalosporins are associated with higher risk for anaphylaxis, we performed sensitivity analyses excluding IV CALs.^6,7,9^

The eMethods, eTable, and eFigure in Supplement 1 provide more information. Data analysis was performed with Stata 18.0 (StataCorp LLC).

## Results

Among 121 eligible patients (median [IQR] age, 49.0 [33.0-67.0] years; 104 females [86.0%]), 23 (19.0%) had verified cephalosporin allergy (20 with a positive skin testing result, 1 with a positive immediate challenge, and 2 with benign delayed morbilliform rash) (Table 1). CEPH-FAST and Vanderbilt criteria could be scored using allergists’ documentation in 120 (99.2%) and 106 (87.6%) patients, respectively, compared with only 9 patients (7.4%) for Urticaria 1-1-1.

**Table 1.** Demographic Characteristics, Medical History, and Testing Results of Patients

Characteristic	Patients, No. (%) (N = 121)
Age, median (IQR), y	49.0 (33.0-67.0)
Sex	
Female	104 (86.0)
Male	17 (14.0)
Race and ethnicity^a^	
Asian	3 (2.5)
Black or African American	4 (3.3)
Hispanic or Latinx	4 (3.3)
White	107 (88.4)
Other or unknown^b^	7 (5.8)
Primary language not English	1 (0.8)
Medical and allergy history^c^	
Charlson comorbidity index, median (IQR)	2.0 (0-4)
Pregnant at time of initial allergist visit	24 (19.8)
Pregnant at time of cephalosporin challenge	6 (5.0)
Asthma	54 (44.6)
Atopic dermatitis	21 (17.3)
Allergic rhinoconjunctivitis	65 (53.7)
Chronic urticaria	15 (12.3)
Food allergy	30 (24.8)
Multiple (≥2) drug allergy labels	100 (82.6)
Implicated cephalosporin	
Cefaclor	17 (14.0)
Cefazolin	19 (15.7)
Cefprozil	10 (8.3)
Ceftriaxone	4 (3.3)
Cefuroxime	14 (11.5)
Cephalexin	45 (37.2)
Unspecified	17 (14.0)
Multiple cephalosporin allergy labels	14 (11.5)
Cephalosporin index reaction history^c^	
Anaphylaxis	22 (18.2)
Perioperative anaphylaxis specifically	16 (13.2)
Angioedema	7 (5.8)
Nonurticarial rash	43 (35.5)
Urticarial rash	38 (31.4)
Remote: >5 y prior	84 (69.4)
Severe cutaneous adverse reaction	2 (1.6)
CEPH-FAST, Vanderbilt criteria, Urticaria 1-1-1 score documented in allergists’ clinical note	0
Risk stratification tool: documentation sufficient to score^d^	
CEPH-FAST	120 (99.2)
Urticaria 1-1-1	9 (7.4)
Vanderbilt criteria	106 (87.6)
Testing characteristics and results	
Testing data completion date, median (IQR)	January 18, 2023 (May 19, 2021, to March 4, 2024)
Positive skin prick or intradermal test	20 (16.5)
Positive immediate challenge following negative skin test	1 (0.8)
Positive direct cephalosporin challenge	0
Benign delayed morbilliform rash following challenge	2 (1.6)

^a^ Race and ethnicity were self-reported. These data were included in this analysis to understand the sociodemographic background of patients who underwent cephalosporin allergy evaluation.

^b^ Other race referred to race not listed in the demographics module of the electronic health record.

^c^ History was obtained via medical record review of allergist notes, other clinician notes, and the electronic health record problem list.

^d^ Indicates whether each tool could be scored based on index reaction descriptions in allergist notes.

CEPH-FAST and Vanderbilt criteria showed specificities of 84.5% (95% CI, 75.8%-91.1%) and 88.2% (95% CI, 79.4%-94.2%) and NPVs of 94.3% (95% CI, 87.1%-98.1%) and 96.2% (95% CI, 89.2%-99.2%), respectively (Table 2). Both tools showed good discriminatory ability (AUROC, CEPH-FAST: 0.81 [95% CI, 0.72-0.91]; Vanderbilt criteria: 0.87 [95% CI, 0.79-0.95]). When excluding IV CALs, the NPV of CEPH-FAST increased slightly, but sensitivity decreased for both CEPH-FAST (78.3% [95% CI, 56.3%-92.5%] to 33.3% [95% CI, 4.3%-77.7%]) and Vanderbilt criteria (85.7% [95% CI, 63.7%-97.0%] to 25.0% [95% CI, 0.6%-80.6%]).

**Table 2.** External Validation of CEPH-FAST and Vanderbilt Criteria for Identifying Positive Test Results in Patients Undergoing Skin Testing and/or Cross-Reactive Cephalosporin Challenge

	CEPH-FAST (n = 120)	CEPH-FAST excluding IV cephalosporin allergy labels (n = 94)	Vanderbilt criteria (n = 106)	Vanderbilt criteria excluding IV cephalosporin allergy labels (n = 81)
Positive test result prevalence in cohort, No. (%)	23 (19.2)	6 (6.4)	21 (19.8)	4 (4.9)
Patients with low risk, No. (%)	87 (72.5)	83 (88.3)	78 (73.6)	74 (91.3)
Positive test result, No. (%)	5 (5.7)	4 (4.8)	3 (3.8)	3 (4.1)
Patients with high risk, No. (%)	33 (27.5)	11 (11.7)	28 (26.4)	7 (8.6)
Positive test result, No. (%)	18 (54.5)	2 (18.2)	18 (64.3)	1 (14.3)
Sensitivity (95% CI), %	78.3 (56.3-92.5)	33.3 (4.3-77.7)	85.7 (63.7-97.0)	25.0 (0.6-80.6)
Specificity (95% CI), %	84.5 (75.8-91.1)	89.8 (81.5-95.2)	88.2 (79.4-94.2)	92.2 (83.8-97.1)
PPV (95% CI), %	54.5 (36.4-71.9)	18.2 (2.3-51.8)	64.3 (44.1-81.4)	14.3 (0.4-57.9)
NPV (95% CI), %	94.3 (87.1-98.1)	95.2 (88.1-98.7)	96.2 (89.2-99.2)	95.9 (88.6-99.2)
Positive LR (95% CI)	5.06 (3.03-8.45)	3.26 (0.90-11.84)	7.29 (3.97- 13.38)	3.21 (0.50- 20.68)
Negative LR (95% CI)	0.26 (0.12- 0.56)	0.74 (0.42-1.31)	0.16 (0.06- 0.46)	0.81 (0.46-1.44)
OR (95% CI)	19.68 (6.50-59.13)	4.39 (0.83-24.19)	45.00 (11.75-168.82)	3.94 (0-33.84)
AUROC (95% CI)	0.81 (0.72-0.91)	0.62 (0.41-0.82)	0.87 (0.79-0.95)	0.59 (0.34-0.83)

Abbreviations: AUROC, area under the receiver operating characteristics curve; IV, intravenous; LR, likelihood ratio; NPV, negative predictive value; OR, odds ratio; PPV, positive predictive value.

## Discussion

This study of patients evaluated for CALs showed that, among 3 risk stratification tools, CEPH-FAST and Vanderbilt criteria had similar performance characteristics compared with the original studies,^6,7,8^ with acceptable sensitivity and specificity and robust NPVs. However, we observed a substantial decrease in sensitivity when excluding patients with IV CALs. Compared with oral medications, IV medications are associated with higher risk for anaphylaxis, likely due to differences in bioavailability, allergic reaction physiological function, and sensitization.^1,6,7,9^ In contrast, in both children and adults, verified oral cephalosporin allergy is rare (<2.5% oral CALs).^7,10^ Our findings underline the importance of using these tools alongside a comprehensive allergy history, considering IV CALs’ higher risk (eg, when using CEPH-FAST), and recognizing that these tools are best at identifying patients with low-risk histories for direct challenge (but not cases of true allergy).

Study limitations include the retrospective design; single site setting; and a predominantly female, White sample with atopic comorbidities. Although skin testing is not a guideline-concordant approach to ruling out drug allergy (without a challenge),^1^ not including patients with negative results also introduces potential for bias. Because this study was conducted at a tertiary referral center and excluded patients directly delabeled, it included a sample enriched for true allergy: in a (general) population with lower prevalence, the tools’ NPV may be higher and PPV may be lower. As the verified allergy cases included patients with positive skin testing results (and the diagnostic validity of cephalosporin skin testing has not been well established), false-positive results were possible. Lastly, medication sensitization patterns vary geographically,^9^ which may affect generalizability.

This research builds on prior studies and lends valuable evidence supporting CEPH-FAST and Vanderbilt criteria in evaluating CALs, along with a comprehensive allergy history that considers administration route of the implicated cephalosporin. Future research should validate these tools in other populations and for IV cephalosporins specifically. Multisite trials and/or meta-analyses may also aid in modeling predictive values over a wide range of population characteristics and in creating tools maximizing sensitivity to identify true allergy cases instead of maximizing NPV to identify candidates for direct challenge.

## Notes

Supplement 1.
eMethods

eReferences
**eTable.** Description of Risk Stratification Tools and Scoring Criteria **eFigure.** CONSORT Diagram of Study Participant Selection

Supplement 2.
Data Sharing Statement
