# Supplementary material for: Performance of Risk Stratification Tools for Cephalosporin Allergy Testing
Source: JAMA Netw Open. 2026 Sep 16;9(9):e2634079. doi: 10.1001/jamanetworkopen.2026.34079 (PMC13583662; doi:10.1001/jamanetworkopen.2026.34079)
Supplement: Supplementary file 1 [file jamanetwopen-e2634079-s001.pdf]

Supplemental Online Content

Belmont AP, Valente M, Workineh A, et al. Performance of risk stratification tools for cephalosporin allergy testing. *JAMA Netw Open*. 2026;9(9):e2634079. doi:10.1001/jamanetworkopen.2026.34079

eMethods

eReferences

eTable. Description of Risk Stratification Tools and Scoring Criteria

eFigure. CONSORT Diagram of Study Participant Selection

This supplemental material has been provided by the authors to give readers additional information about their work.

© 2026 Belmont AP et al. *JAMA Netw Open*.

eMethods

Institutional Review Board (IRB)

We received IRB exemption and waiver of informed consent for this cross-sectional study.

Data Collection and Coding

Data Collection from the Electronic Health Record (EHR) including Inclusion of Sociodemographic Characteristics

Potentially eligible patients were identified in July 2025 by the Yale School of Medicine Joint Data Analytic Team (JDAT) through a query identifying patients with (active or deleted) cephalosporin allergy labels in the Yale New Haven Epic™ electronic health record (EHR) who were seen in a Yale School of Medicine-affiliated Allergy/Immunology Clinic and who had allergy testing (determined by CPT codes 95018 and 95076). We included patients with the following cephalosporin allergy labels: Cefadroxil, Cephalexin, Cefazolin, Cefaclor, Cefoxitin, Cefprozil, Cefuroxime, Cefdinir, Cefditoren, Cefixime, Cefotaxime, Cefpodoxime, Ceftazidime, Ceftibuten, Ceftriaxone, Cefepime, Ceftaroline, Ceftolozane, and “cephalosporins” otherwise unspecified. Using this list, the study team performed manual chart review to build a database of patients seen for consultative visits for cephalosporin allergy evaluation.

We performed consecutive sampling of patients evaluated at the Yale Allergy/Immunology Clinic during the pre-defined study (12/1/2014 – 7/21/2025). Using the list provided by JDAT, we performed retrospective chart review to identify patients who met full inclusion criteria (e.g., who underwent cephalosporin skin testing followed by cephalosporin challenge, if appropriate, or direct cephalosporin challenge with the implicated or a cross-reactive cephalosporin) (eFigure). Of note, a cross-reactive cephalosporin refers to a cephalosporin that shares a core structural component implicated in Immunoglobulin E (IgE)-mediated allergy (e.g., the R1 side chain);[1] if a

© 2026 Belmont AP et al. *JAMA Netw Open*.

patient is allergic to a cephalosporin they are expected to have a high risk for a reaction if given a cephalosporin with a similar R1 side chain, referred to as a cross-reactive cephalosporin.[1] We identified cross-reactive cephalosporins using cross-reactivity charts in the Joint Task Force of the American Academy of Allergy, Asthma, and Immunology (AAAAI) and the American College of Allergy & Immunology (ACAI) “Drug Allergy: A 2022 Practice Parameter Update.”[1] We performed manual chart review on all patients to identify receipt of cephalosporins after testing. Of note, if a patient had undergone skin testing and/or a challenge with a non-cross-reactive cephalosporin (but not a cross-reactive cephalosporin), but later received a cross-reactive cephalosporin, we included such patients (after careful review to ensure they had follow up in the system for at least three months after the prescription). Consistent with prior studies, tolerance was adjudicated by manual chart review of visits within one month of the cephalosporin prescription, as well as the allergy module.[2] Patients were not included if they never received a cross-reactive cephalosporin (even despite negative skin testing or receipt of a non-cross-reactive cephalosporin), as the guideline-recommended standard of care to determine tolerance is a drug challenge (not skin testing alone).[1] Likewise, similar to the original CEPH-FAST study, for patients in which the implicated cephalosporin was unknown, a challenge to any cephalosporin was considered acceptable.[3]

We collected all study data via retrospective manual chart review (MV, AW, SN, AB) and stored it in REDCap. Data management, supervision, data analyses, and review of all allergy records was performed by a physician board-certified allergist (AB). We included race and ethnicity data to understand the sociodemographic background of patients who underwent cephalosporin allergy evaluation. Investigators obtained race and ethnicity data from the electronic health record. Of note, we determined the implicated cephalosporin and index reaction through review of the allergist’s notes. In the event of a discrepancy between the implicated cephalosporin in

© 2026 Belmont AP et al. *JAMA Netw Open*.

the allergy module and that described by the allergist, we considered the allergist's history gold-standard, consistent with existing literature showing this as the most reliable manner to obtain drug allergy reaction data.[4]

Consistent with approaches used in prior literature, reactions were scored (as able) based on scoring criteria in the original CEPH-FAST, Vanderbilt criteria, and Urticaria 1-1-1 studies (eTable).[5] We recorded whether each tool could be scored based on documentation in the allergists' notes. Acknowledging the limitation that the ability to score reactions with each tool may partly reflect local documentation practice, as well as knowledge of the tool itself (which could in turn favor tools already in use), we also recorded whether allergists documented mention and/or use of each tool. We performed scoring with each tool prior to reviewing testing results; however this was not blinded in a standardized manner.

Cephalosporin testing was performed in alignment with drug allergy guidelines.[1] Patients underwent cephalosporin skin testing followed by a cephalosporin challenge, or a direct cephalosporin challenge (with no preceding skin testing).[1] The testing strategy was determined by the treating allergist at an initial consultative visit. Skin testing included skin prick testing and intradermal testing with intravenous cephalosporin agents at published non-irritating concentrations.[1] There was no standard skin testing panel, and consistent with Yale New Haven Hospital guidelines, oral cephalosporins were not formulated for skin testing (as they were not considered sterile). If skin testing was positive, no cephalosporin challenge was performed to a cross-reactive cephalosporin. Cephalosporin challenges were completed using a two-step challenge protocol (e.g., approximately 10% of the target dose, followed by a 30-minute observation window, then 90% of the target dose, followed by a 60-minute observation window).

© 2026 Belmont AP et al. *JAMA Netw Open*.

As per inclusion criteria detailed above, only patients who had a challenge with a cross-reactive cephalosporin were included. All patients were evaluated by board-certified or eligible allergists.

Data Analysis

We performed descriptive analyses. We also assessed the proportion of patients for which we could use each risk stratification tool. If a tool was able to be used for > 80 patients, we calculated the following among patients that had a score: sensitivity, specificity, negative predictive value, positive predictive value, negative likelihood ratio, and positive likelihood ratio (with confidence intervals). To understand the diagnostic ability of each tool, we also calculated the area under the receiver operating characteristics curve. Missing data were not included in the analyses. We performed data analysis using STATA 18.0 (StataCorp, College Station, Texas) with the assistance of the diagt software package.[6]

© 2026 Belmont AP et al. *JAMA Netw Open*.

eReferences (see full reference list in main text)

1. Khan DA, Banerji A, Blumenthal KG, et al. Drug allergy: A 2022 practice parameter update. *J Allergy Clin Immunol*. Dec 2022;150(6):1333-1393. doi:10.1016/j.jaci.2022.08.028
2. Macy E, McCormick TA, Adams JL, et al. Association between removal of a warning against cephalosporin use in patients with penicillin allergy and antibiotic prescribing. *JAMA Network Open*. 2021;4(4):e218367-e218367.
3. Cox F, Vogrin S, Sullivan R, et al. Development and validation of a cephalosporin allergy clinical decision rule. *Journal of Infection*. 2025;90(6):106495.
4. Novotny S, Elmansy L, Kwah J, et al. High-risk penicillin reaction flags in the medical record. *JAMA Network Open*. 2025;8(12):e2549081.
5. Su C, Belmont A, Liao J, Kuster JK, Trubiano JA, Kwah JH. Evaluating the PEN-FAST Clinical Decision-making Tool to Enhance Penicillin Allergy Delabeling. *JAMA Intern Med*. Jun 20 2023;doi:10.1001/jamainternmed.2023.1572
6. Seed P. DIAGT: Stata module to report summary statistics for diagnostic tests compared to true disease status. 2010;
7. Koo G, Yu R, Phillips EJ, Stone Jr CA. Retrospective stratification of cephalosporin allergy label risk using validated penicillin allergy frameworks. *The Journal of Allergy and Clinical Immunology: In Practice*. 2022;10(9):2472-2475. e1.
8. Sabato V, Gaeta F, Valluzzi RL, Van Gasse A, Ebo DG, Romano A. Urticaria: the 1-1-1 criterion for optimized risk stratification in β-lactam allergy delabeling. *The Journal of Allergy and Clinical Immunology: In Practice*. 2021;9(10):3697-3704.

© 2026 Belmont AP et al. *JAMA Netw Open*.

eTable: Description of Risk Stratification Tools and Scoring Criteria

**Tools**	**Scoring**	**Negative predictive value**
CEPH-FAST[3]	• Recent reaction ≤5 years ago − 2 points<br>• Anaphylaxis, angioedema, or severe cutaneous adverse reactions − 2 points<br>• Treatment required − 1 point<br><br>*Unknown timing or treatment variables did not receive a point*	Total score ≤ 2 – NPV 94.3%[3]
Vanderbilt criteria[7]	<u>Highest risk:</u><br>• Severe cutaneous adverse reaction (e.g., Stevens-Johnson syndrome/Toxic epidermal necrolysis, drug reaction with eosinophilia and systemic symptoms, acute generalized exanthematous pustulosis)<br>• Serum sickness<br>• Immune-mediated kidney or liver injury<br>• Reaction to an intravenously administered cephalosporin<br><br><u>High risk:</u><br>• Anaphylaxis within the past 5 years<br>• Within 1 hour of cephalosporin administration, the patient developed: diffuse hives, angioedema, shortness of breath, wheezing or coughing, hypotension, weak pulse, loss of consciousness, diarrhea, or vomiting<br>• Reaction to an IV- administered cephalosporin<br><u>Low risk:</u><br>• Isolated urticaria more than 5 years ago<br>• Self-limited cutaneous rash at any point in the past (without organ involvement)<br>• Gastrointestinal symptoms only<br>• Remote childhood reaction with limited details<br>• Family history of cephalosporin allergy only<br>• Known tolerance of a cephalosporin since the original reaction occurred<br>• Other nonallergy symptoms<br>• Reaction to an orally administered cephalosporin	Low risk history – NPV 98.5%[7]
Urticaria 1-1-1[8]	• Time interval between last administered dose and onset of eruption ≤ 1 hour − 1 point<br>• Urticarial eruption appeared after 1 dose − 1 point<br>• Duration of eruption ≤ 1 day − 1 point<br>*Original study: Patients unable to correctly report 1 or more criteria were not included. We did not score patients when all three items were not recorded.*	Total score ≤ 2 − NPV 80%[8]

IV, intravenous; NPV, negative predictive value

© 2026 Belmont AP et al. *JAMA Netw Open*.

eFigure: CONSORT Diagram of Study Participant Selection

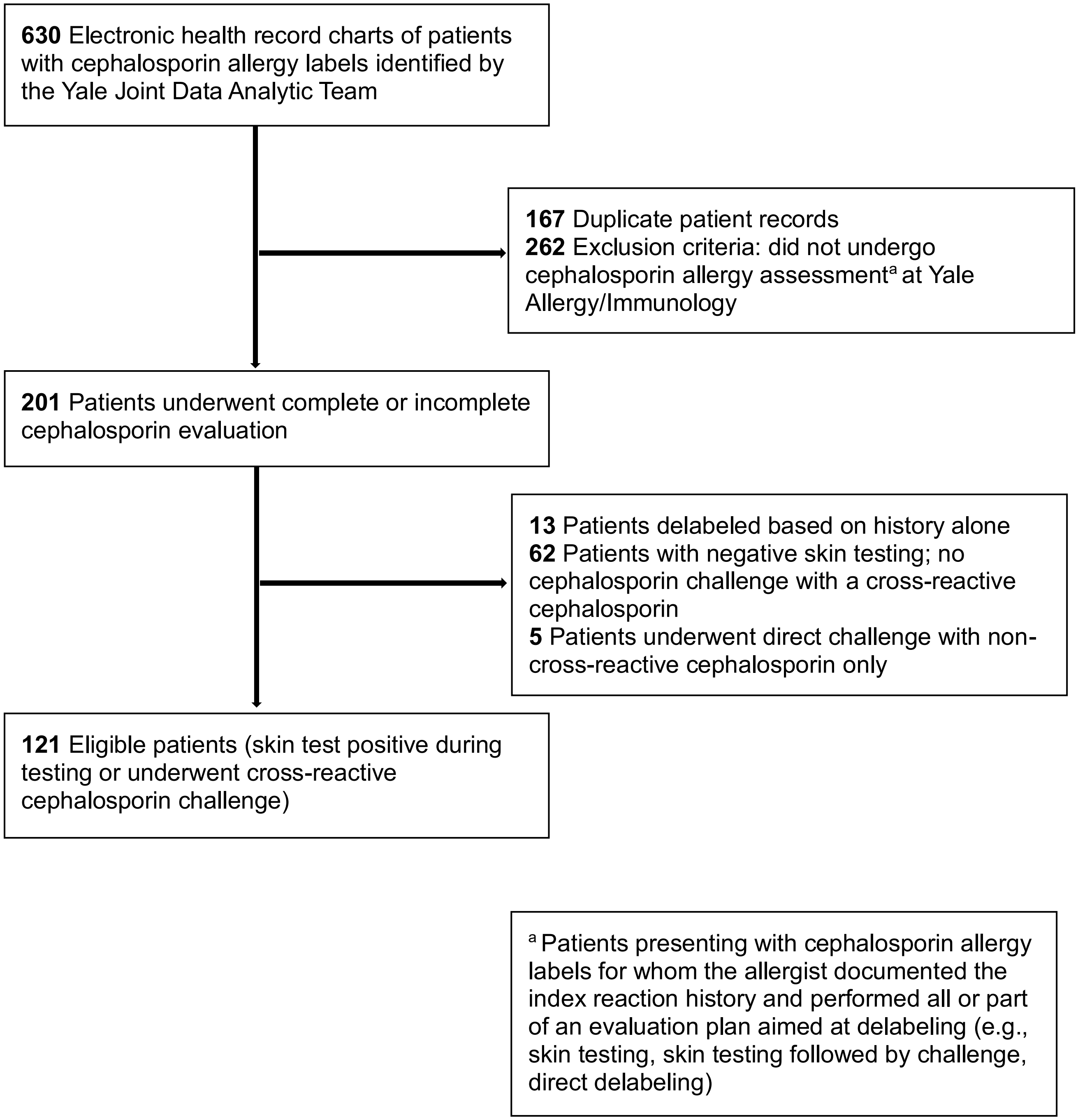

© 2026 Belmont AP et al. *JAMA Netw Open*.
