# Supplementary material for: Performance of Risk Stratification Tools for Cephalosporin Allergy Testing
Source: JAMA Netw Open. 2026 Sep 16;9(9):e2634079. doi: 10.1001/jamanetworkopen.2026.34079 (PMC13583662; doi:10.1001/jamanetworkopen.2026.34079)
Supplement: Supplementary file 2 [file jamanetwopen-e2634079-s002.pdf]

# Data Sharing Statement

Belmont. Performance of Risk Stratification Tools for Cephalosporin Allergy Testing. *JAMA Netw Open*. Published September 16, 2026. doi:10.1001/jamanetworkopen.2026.34079

**Data**
**Data available:** Yes
**Data types:** Deidentified participant data
**How to access data:** This will be available upon request to the PI.
**When available:** With publication

**Supporting Documents**
**Document types:** None

**Additional Information**
**Who can access the data:** Researchers whose proposed use of the data has been approved.
**Types of analyses:** For approved research purposes only.
**Mechanisms of data availability:** with investigator support.
